# *IGF2BP2* and *IGFBP3* Genotypes, Haplotypes, and Genetic Models Studies in Polycystic Ovary Syndrome

**DOI:** 10.1002/jcla.25021

**Published:** 2024-03-11

**Authors:** Fatemeh Govahi Kakhki, Saman Sargazi, Farzaneh Montazerifar, Mahdi Majidpour, Atena Karajibani, Mansour Karajibani, Marzieh Ghasemi

**Affiliations:** ^1^ Department of Nutrition, School of Medicine Zahedan University of Medical Sciences Zahedan Iran; ^2^ Cellular and Molecular Research Center, Research Institute of Cellular and Molecular Sciences in Infectious Diseases Zahedan University of Medical Sciences Zahedan Iran; ^3^ Department of Clinical Biochemistry, School of Medicine Zahedan University of Medical Sciences Zahedan Iran; ^4^ Pregnancy Health Research Center Zahedan University of Medical Sciences Zahedan Iran; ^5^ Clinical Immunology Research Center, Zahedan University of Medical Sciences Zahedan Iran; ^6^ Department of Biology University of Sistan and Baluchestan Zahedan Iran; ^7^ Health Promotion Research Center Zahedan University of Medical Sciences Zahedan Iran; ^8^ Moloud Infertility Center, Ali Ibn Abitaleb Hospital Zahedan University of Medical Sciences Zahedan Iran

**Keywords:** gene polymorphism, *IGF2BP2*, *IGFBP3*, polycystic ovarian syndrome

## Abstract

**Background:**

Insulin resistance has been correlated with the genetic diversity within the insulin‐like binding proteins genes. Moreover, insulin resistance is one of the key characteristics of the widespread reproductive endocrine condition known as polycystic ovarian syndrome (PCOS). Hence, this study is aimed to determine the association between *IGFBP3* and *IGF2BP2* gene variants and PCOS risk.

**Methods:**

A total of 300 subjects (150 PCOS cases diagnosed based on Rotterdam ESHRE/ASRM consensus criteria and 150 healthy subjects) were recruited in this case–control cross‐sectional study. Tetra‐primer amplification refractory mutation system polymerase chain reaction (ARMS‐PCR) was used for genotyping rs11705701, whereas genotyping of rs1470579 and rs2854744 was done employing PCR‐restriction fragment length polymorphism (PCR‐RFLP) technique.

**Results:**

The CC and AA+AC genotypes of rs1470579 conferred an increased risk of PCOS in our population. Regarding the rs2854744, an increased risk of PCOS was observed under the codominant homozygous (TT vs. GG) model by 2.54 fold. The C allele of rs1470579 and T allele of rs2854744 enhanced PCOS risk by 1.97 and 1.46 folds, respectively. Haplotype analysis showed that the A_rs1470579_A_rs11705701_ haplotype conferred a decreased risk of PCOS (odds ratio = 0.53, 95% confidence interval = 0.34–0.83, *p* = 0.006). The AC/GG/GT, AA/GA/GT, AC/GA/GG, and AC/GA/GT genotype combinations of rs1470579/rs11705701/rs2854744 were associated with a decreased risk of the disease.

**Conclusions:**

*IGF2BP2* rs1470579 and *IGFBP3* rs2854744 enhanced PCOS susceptibility in a Southeastern Iranian population. Further investigation involving larger cohorts representing diverse ethnic backgrounds is needed to confirm the current findings.

## Graphical abstract

Analysis of the association between *IGF2BP2* and *IGFBP3* polymorphisms and the risk of polycystic ovary syndrome (PCOS) was carried out on a total of 300 subjects using both ARMS‐PCR and PCR‐RFLP methods. Visualization of gel electrophoresis patterns showed a positive association between rs1470579 and PCOS and a negative association between rs2854744 and susceptibility to this endocrine disease.

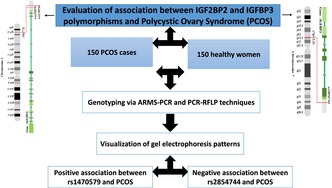

## 1 Introduction

Polycystic ovarian syndrome (PCOS) is a commonly observed endocrine disorder, particularly in women of childbearing age, which significantly affects menstrual and fertility functions and can have adverse health consequences throughout a woman's lifespan [1]. It has been approximated that the global percentage of this condition ranges from 5% to 10%. In addition, there have been significant variations in the rates of PCOS incidence among various Asian populations. For example, the prevalence is recorded at 6.3% in Sri Lanka, 5.6% in China, 5.3% in Thailand, and 15.2% in Iran [2, 3, 4]. A meta‐analysis study by Tehrani et al. in 2011 reported that although PCOS is not prevalent in Iran, it appears that the intensity of PCOS symptoms increases with age due to adipose tissue accumulation. Despite this, several studies have found a positive correlation between clinical symptoms and the prevalence of PCOS, and our study confirmed this. PCOS prevention is important because of its symptoms, as well as its risks of cardiovascular disease and infertility [5]. Similarly, Mehrabian and colleagues conducted a study in the same year that estimated PCOS prevalence at 7.92% according to the AES criteria and 15.2% according to Rotterdam criteria [6].

Women with PCOS experience symptoms from utero onward, beginning in adolescence in those genetically predisposed and persisting throughout their reproductive years. PCOS cases, particularly after menopause, can also be prone to metabolic disorders such as hypertension, diabetes, cardiovascular disease, and others [7]. As well as having an increased risk of miscarriage [8], gestational diabetes, and preeclampsia, PCOS can cause infertility throughout a pregnant woman's fertile period [9]. It is, therefore, imperative to diagnose early, followed by diligent follow‐up, and take steps to reduce the chances of such complications. Evidence shows that genetics and environment play a role in PCOS [10]. Up to 2020, over 200 susceptibility genes, including *17‐Hydroxysteroid dehydrogenase type 5* (*HSD17B5*), *Calpain‐10* (*CAPN10*), *Fibrillin 3* (*FBN3*), *cytochrome P450 family 11 subfamily A member 1* (*CYP11A1*), *Follistatin, and insulin receptor* (*INSR*), have been associated with PCOS in some studies [11].

**SCHEME 1:**
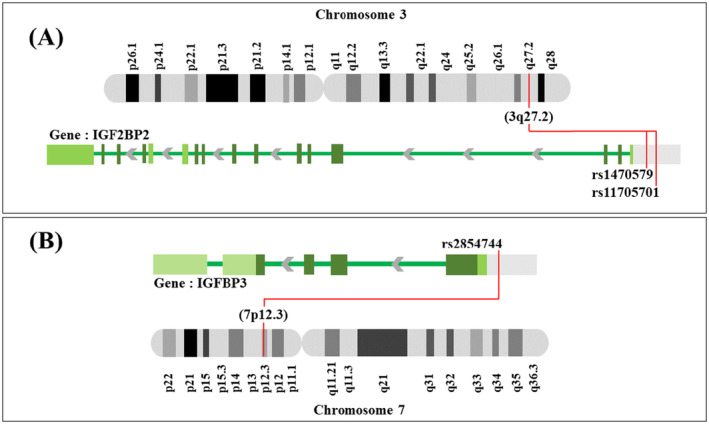
Location of *IGF2BP2* and *IGFBP3* polymorphirms located on chromosome 3 (A, 3p13.1) and 7 (B, 7p13.1).

Known as insulin‐like growth factor II (IGF‐II) mRNA‐binding protein 2 (IGF2BP2), it binds to the crucial growth and insulin signaling molecule IGF‐II. IGF2BP2 is encoded by the *IGF2BP2* gene located on chromosome 3q27 [12, 13]. PCOS is characterized by insulin resistance; therefore, SNPs linked to the insulin signaling pathways may contribute to the development of PCOS's clinical features [14]. Two SNPs, the rs11705701 G/A (GRCh38.p12, minor allele frequency [MAF] = 0.49) and rs1470579 A/C (GRCh37.p13, MAF = 0.45), have already been correlated to T2DM susceptibility in other races [15]. Scheme 1 shows the location of *IGF2BP2* rs11705701 and rs1470579 polymorphisms on chromosome 3q27.

Many investigations have shown that IGF‐II is abnormally expressed in female reproductive tumors [16]. A study in India was conducted on the *IGF2BP2* gene to investigate the link between T2DM genes and susceptibility to PCOS. They found that after adjusting for body mass index (BMI), a significant association between *IGF2BP2* (rs1470579) and PCOS, which probably revealed the role of BMI as a possible confounder in masking the protective role of this gene against manifestations of PCOS. Therefore, Genes related to adipocyte differentiation and insulin pathways may play a role in the pathogenesis of PCOS [17].

Additionally, IGFBP3 is required for normal growth and development, like its counterparts IGF2BP2 and IGF mRNA‐binding protein 3 (IMP‐3). As a result of its attachment to the 5′UTR of the IGF‐II leader 3 mRNA, they have a profound influence on post‐transcriptional processes [12, 13]. A relatively high expression of *IGFBP3* is also found in the ovaries [18].

The effects of SNPs on multifactorial diseases differ between racial and ethnic groups. There has been no investigation of the frequency of *IGF2BP2* and *IGFBP3* polymorphisms, in southeast Iranian women with PCOS. Hence, we examined rs11705701, rs1470579, and rs2854744 to explore the association of these SNPs with disease risk.

## 2 Materials and Methods

### 2.1 Subjects and Sample Collection

A total of 300 subjects were enrolled in the current cross‐sectional case–control study (150 PCOS cases and 150 controls) and referred to the Boo‐Ali Hospital in Zahedan, Iran, between December 1, 2022, and September 1, 2023. Patients were diagnosed with PCOS based on a consensus criteria developed by Rotterdam American Society for Reproductive Medicine (ASRM)/European Society of Human Reproduction and Embryology (ESHRE) [19]. We excluded women suffering from endocrine and systemic diseases, cancer, premature ovarian failure, virilizing and autoimmunity defects, liver disease, and having a family relationship. Women with regular menstrual cycles (28–32 days) and without endocrine disorders except obesity (BMI >30 kg/m^2^) and overweight (BMI >25 kg/m^2^) were used as control subjects. The controls with biochemical hyperandrogenism were excluded from the study. There was no abnormality in healthy women's medical history, biochemical tests, or physical examinations. Anthropometric characteristics, such as height, weight, and waist circumference (WC), were calculated as previously discussed [20]. The clinical and demographic characteristics of the two groups are summarized in Table 1.

**TABLE 1:** Clinical and demographic characteristics of PCOS patients and controls.

Parameter evaluated	Reference range	PCOS (mean ± SD)	Controls (mean ± SD)	*p* Value^a^
Age (years)		27.52 ± 5.19	28.93 ± 6.01	0.055
BMI (kg/m^2^)		28.85 ± 4.76	25.43 ± 3.86	**<0.001**
Underweight (BMI < 18.5)		3	6	—
Ideal (18.5 < BMI < 24.9)		22	45	—
Overweight (25 < BMI < 30)		71	86	—
Obese (BMI > 30)		54	13	—
WC (cm)	Ideal: less than 80	102.00 ± 13.07	94.51 ± 10.63	**<0.001**
FBS (mg/dL)	76–99^b^	97.17 ± 16.44	93.86 ± 7.04	0.068
Prolactin (μg/L)	Less than 25^b^	29.77 ± 11.66	15.74 ± 4.59	**<0.001**
Free testosterone (pg/mL)	50–200^b^	2.89 ± 1.17	7.52 ± 2.46	**<0.001**
DHEA‐S (μg/dL)	35–430^b^	92.74 ± 13.87	213.39 ± 20.63	**<0.001**
TC (mg/dL)	Less than 200^b^	200.45 ± 16.36	184.72 ± 19.91	**<0.001**
TG (mg/dL)	Less than 150^b^	134.93 ± 50.19	124.16 ± 15.13	**0.002**
LDL‐c (mg/dL)	Less than 100^b^	110.35 ± 16.20	105.44 ± 7.30	0.089
HDL‐c (mg/dL)	35–80^b^	41.04 ± 7.48	38.74 ± 3.14	**0.001**
Systolic pressure (mmHg)	Less than 120	131.2 ± 9.0	116.4 ± 11.0	**<0.001**
Diastolic pressure (mmHg)	Less than 80	98.2 ± 14.9	74.4 ± 6.4	**<0.001**

*Note: p* < 0.05 was considered statistically significant (bold *p*‐values).

Abbreviations: BMI, body mass index; DHEA‐S, dehydroepiandrosterone sulfate; FBS, fasting blood sugar; HDL‐c, high‐density lipoprotein‐c; LDL‐c, low‐density lipoprotein‐c; PCOS, polycystic ovarian syndrome; TC, total cholesterol; TG, triglyceride; WC, waist circumference.

^a^ Mann–Whitney test.

^b^ Reference ranges were considered according to the acceptable ranges of our assay kits.

### 2.2 Blood Sampling and Biochemical Analyses

After fasting for at least 12 h, a total of 5 mL venous blood was drawn from control groups and PCOS patients. Three milliliters of venous whole blood were collected into tubes containing ethylene diamine tetraacetic acid (EDTA) for DNA extraction. In addition, 3 mL of blood was collected for biochemical analysis, including measurements of serum lipid profiles (including triglycerides [TG], low‐density lipoprotein‐c [LDL‐c], high‐density lipoprotein‐c [HDL‐c], and total cholesterol [TC]) and/or fasting blood sugar (FBS). Such biochemical parameters were assessed using Pars Azmoon kits (Tehran, Iran). An enzyme‐linked immunosorbent assay (ELISA) protocol was used to assay serum levels of prolactin (assay kit provided by Pishtaz Teb Diagnostics, Tehran, Iran), free testosterone, and dehydroepiandrosterone sulfate (DHEA‐S) (assay kit provided by Eagle Biosciences, USA).

### 2.3 SNP Selection and Primer Design

The rs11705701, rs1470579, and rs2854744 SNPs in *IGF2BP2* and *IGFBP3* genes, whose MAFs are 0.49, 0.45, and 0.46, respectively, were chosen due to their relatively high frequencies among populations, as reported by the 1000 Genome Project. Table S1 shows the primer pairs designed using Gene Runner and synthesized by Genfanavaran Co (Tehran, Iran).

### 2.4 DNA Extraction and Genotyping

Salting out was used to isolate DNA using a simple extraction method [21]. Gel electrophoresis and spectrophotometric analysis of the A260/280 ratio were used to determine DNA quality and quantity, respectively. *IGF2BP2* rs1470579 and *IGFBP3* rs2854744 polymorphisms were genotyped using polymerase chain reaction‐restriction fragment length polymorphism (PCR‐RFLP), while *IGF2BP2* rs11705701 was genotyped using amplification refractory mutation system PCR (ARMS‐PCR). The PCR products were subsequently digested using *Fok1* and *Hha1* restriction enzymes. Table S2 shows PCR conditions for genotyping SNPs. As a final step, the PCR bands were visualized on 2% agarose gels (Figure S1). The genotyping accuracy of 100% was achieved by randomly genotyping 20% of the samples.

### 2.5 Statistical Analysis

Data were analyzed by SPSS v16 software. The Kolmogorov–Smirnov test was used to assess the normality of the distribution. Descriptive statistics, including frequency percentage, frequency, standard deviation, and mean, were used to describe quantitative variables. A single sample *t*‐test, Mann–Whitney–Wilcoxon and Pearson chi‐square tests were used as appropriate to compare continuous variables between cases and controls. Independent sample *t*‐tests and chi‐square tests were used where appropriate to analyze the quantitative data. The risk of PCOS was also estimated using a 95% confidence interval (CI) as well as an odds ratio (OR) and 95% CI. Polymorphisms of *IGF2BP2* and *IGFBP3* were analyzed using binary logistic regression analysis. A pair of odds calculates the OR for each genotype and/or allele after determining each SNP's major and minor alleles. An OR can be estimated simply by combining data using the two‐by‐two contingency method. Then, dominant, codominant, recessive, and over‐dominant models are calculated as described by Horita et al. [22]. Possible correlations between PCOS risk and the cases' clinical and demographic features were analyzed using logistic regression. In the regression analysis, firstly, the variables' raw (unadjusted) relationship with the genotypes was checked, and then, the assessed correlation was adjusted for age and BMI. As described by Goodman et al. [23], SNP Interaction Analysis was done to determine whether genotypes of each SNP appeared regularly, alone or in combination with other genotypes of the other examined SNPs. Linkage disequilibrium (LD) and population haplotype patterns were analyzed using initial genotyping frequencies via the online SHEsis software [24, 25] (accessible on the website http://analysis.bio‐x.cn/), and a plot was drawn. The software calculated Lewontin's *D*′ (|*D*′|) [26] between each pair of genetic markers.

### 2.6 In Silico Analysis

Promo v.3.0.2, a virtual laboratory developed by ALGGEN at the Technical University of Catalonia, was used in our study to identify putative transcription factor binding sites in DNA sequences (TFBS) [27, 28]. To accomplish this, TFBS predictions for *IGF2BP2* rs11705701 and *IGFBP3* rs2854744 promoter variations were performed using the TRANSFAC database v.8.3. (accessible at https://alggen.lsi.upc.es). In this regard, Homo sapiens factors and their connection recognition sites were chosen. In order to identify our current factor and site species, the first “Current factor's species or group” was placed on the “human, Homo sapiens.” In order to calculate the maximum deviation between the actual binding site and the predicted one, we determined that the dissimilarity margin for factors predicted within that margin was 5%, and we inserted the gene sequences related to the promoter positions extracted from the NCBI database.

## 3 Results

### 3.1 Clinical and Demographic Findings

The average BMI in PCOS patients and the control group were 28.85 ± 4.76 and 25.43 ± 3.86, respectively (*p* < 0.001). Regarding age, there was no significant difference between the two groups (*p* = 0.055). There were also significant differences in demographic and laboratory parameters between the two groups, as well as systolic and diastolic blood pressure, prolactin, HDL‐c, WC, TC, and TG (*p* < 0.001). As shown in Table 2, the enrolled patients have shown biochemical signs of hyperandrogenism, indicated by significant differences between free testosterone and DHEA‐S levels between the studied groups (*p* < 0.001).

**TABLE 2:** Selected SNP's characteristics.

RS ID ref/alt sequence	Position	Chr no. cyto‐band	Gene symbol	Function (variant type)	Our population frequencies (ƒ)					Other population frequencies (ƒ)				
					Population	Allele count^a^	Allele number	Allele frequency^b^	Ref	Population	Allele count^a^	Allele number	Allele frequency^b^	Ref
rs1470579 A/C	185811292	Chr 3 3q27.2	*IGF2BP2*	Intronic (SNV)	Iran	200	594	C = 0.337	—	China	129	378	C = 0.341	[29]
										Iran	212	536	C = 0.395	[30]
										Taiwan	1231	5094	C = 0.242	[31]
										China	1092	4482	C = 0.244	[32]
										Lebanon	804	2298	C = 0.350	[33]
rs11705701 G/A	185826521	Chr 3 3q27.2	*IGF2BP2*	2KB upstream promoter region (SNV)	Iran	176	586	A = 0.300	—	Taiwan	1090	5094	A = 0.214	[31]
										Poland	352	822	A = 0.428	[34]
										China	648	2978	A = 0.217	[35]
										United States	473	1434	A = 0.330	[36]
										Russia	2334	5782	A = 0.404	[37]
rs2854744 G/T	45921476	Chr 7 7p12.3	*IGFBP3*	2KB upstream promoter region (SNV)	Iran	207	598	T = 0.346	—	Iran	488	1200	T = 0.407	[38]
										Beijing	127	484	G = 0.262	[39]
										Brazil	246	506	T = 0.486	[40]
										Iran	491	1056	T = 0.465	[41]
										China	879	4850	T = 0.181	[42]

Abbreviations: Chr, chromosome; KB, kilo base pair; MAF, minor allele frequency; RS, reference SNP (single nucleotide polymorphism); SNV, single nucleotide variation.

^a^ The number of chromosomes in the population that contain the alternate (nonreference) allele.

^b^ Allele count divided by allele number.

### 3.2 Genetic Association Studies

After adjustment for BMI and age, the CC genotypes of rs1470579 were significantly associated with an increased PCOS risk (OR = 3.57, 95% CI [1.63–7.84], *p* = 0.002). We have also noticed an increased risk of PCOS under this variant's dominant (AA+AC vs. CC) model (OR = 2.08, 95% CI [1.28–3.38], *p* = 0.003). Similarly, the rs2854744 increased the risk of PCOS under the codominant homozygous mode of inheritance (TT vs. GG, OR = 2.54, 95% CI [1.09–5.87], *p* = 0.030). The C allele of rs1470579 (OR = 1.57, 95% CI [1.37–2.84], *p* < 0.001) and T allele of rs2854744 (OR = 1.46, 95% CI [1.03–2.05], *p* = 0.031) enhanced PCOS risk by 1.97 and 1.46 folds, respectively. Meanwhile, estimated ORs indicated that rs11705701 polymorphism is not associated with PCOS risk (*p* > 0.05) (Table S3). Table 2 has provided information about the location, function, and the MAFs of the studied SNPs in our population compared with other populations from china, United States, Russia, Brazil, Taiwan, etc.

### 3.3 Haplotype and Interaction Analyses

Haplotype analysis was performed between rs1470579 and rs11705701. Based on our findings, the A_rs1470579_A_rs11705701_ haplotype conferred a decreased risk of PCOS, compared to the A_rs1470579_G_rs11705701_ genotype (OR = 0.53, 95% CI [0.34–0.83], *p* = 0.006) (Table 3). In this connection, no LD was found between the *IGF2BP2* variations (Figure S2). Moreover, the AC/GG/GT, AA/GA/GT, AC/GA/GG, and AC/GA/GT genotype combinations of rs1470579/rs11705701/rs2854744 were associated with a decreased risk of the disease in our population (OR < 0.3, *p* = 0.002, <0.001, <0.001, and 0.014, respectively) (Table 4).

**TABLE 3:** Haplotype analysis of *IGF2BP2* SNPs on PCOS risk.

rs1470579	rs11705701	PCOS (%)	Control (%)	OR (95% CI)	*p* Value
A	G	141 (48.9)	140 (47.7)	1 [reference]	
C	G	73 (25.3)	54 (18.3)	1.34 (0.87–2.05)	0.172
A	A	39 (13.6)	73 (24.7)	0.53 (0.34–0.83)	**0.006**
C	A	35 (12.2)	27 (9.3)	1.29 (0.74–2.24)	0.372

*Note:* Bonferroni correction was applied, and *p* < 0.025 was considered statistically significant (bold *p*‐value).

Abbreviations: CI, confidence interval; OR, odds ratio; PCOS, polycystic ovarian syndrome.

**TABLE 4:** Interaction analysis of the studied SNPs on PCOS risk.

rs1470579	rs11705701	rs2854744	PCOS (%)	Control (%)	OR (95% CI)	*p* Value
AA	GG	GT	21 (16.4)	15 (11.3)	1 [reference]	
AC	GG	GT	19 (14.8)	14 (10.5)	0.26 (0.11–0.63)	**0.002**
AA	GG	GG	13 (10.2)	18 (13.5)	0.76 (0.35–1.65)	0.489
AA	GA	GT	11 (8.6)	14 (10.5)	0.15 (0.04–0.49)	**<0.001**
AA	GA	GG	2 (1.5)	20 (14.9)	0.74 (0.33–1.69)	0.484
AC	GA	GG	16 (12.5)	5 (3.8)	0.08 (0.02–0.39)	**<0.001**
AC	GG	GG	6 (4.6)	13 (9.8)	1.54 (0.69–3.45)	0.289
AC	GA	GT	11 (8.6)	7 (5.3)	0.23 (0.07–0.79)	**0.014**
CC	GG	GG	7 (5.5)	4 (2.9)	0.75 (0.29–1.98)	0.568
AA	AA	GT	3 (2.3)	6 (4.5)	0.63 (0.21–1.87)	0.404
AC	AA	GT	4 (3.1)	4 (2.9)	0.47 (0.15–1.51)	0.201
CC	GG	GT	5 (3.8)	3 (2.2)	0.18 (0.03–0.93)	0.026
AC	GG	TT	4 (3.1)	1 (0.7)	1.15 (0.38–3.47)	0.804
AC	AA	GG	1 (0.7)	3 (2.2)	0.38 (0.08–1.70)	0.195
AC	GA	GG	1 (0.7)	3 (2.2)	0.25 (0.04–1.39)	0.095
CC	AA	GG	2 (1.5)	1 (0.7)	1.57 (0.28–8.67)	0.605
CC	AA	TT	1 (0.7)	1 (0.7)	1.88 (0.35–10.02)	0.455
AC	AA	TT	1 (0.7)	1 (0.7)	0.94 (0.15–6.01)	0.950
AA	AA	TT	1 (0.7)	1 (0.7)	0.31 (0.03–3.63)	0.333

*Note:* Bonferroni correction was applied, and *p* < 0.016 was considered statistically significant (bold *p*‐value).

Abbreviations: CI, confidence interval; OR, odds ratio; PCOS, polycystic ovarian syndrome.

### 3.4 Association of SNPs With Subject's Clinical‐Demographic Characteristics

A regression model was developed to examine the relationship between SNPs and biochemical‐anthropometric features of the participants. A meaningful difference among the genotypes (AA+AC vs. CC) of rs1470579 was observed in terms of prolactin (*p* < 0.001) and HDL‐c (*p* = 0.018) levels in PCOS patients, along with the FBS (*p* = 0.037) and TC (*p* = 0.007) levels in healthy women. There was also a marked difference between carriers of the GG + GA and AA genotypes of rs11705701 concerning TG (*p* = 0.016) and LDL‐c (*p* < 0.001) levels in the cases (Table 5).

**TABLE 5:** Association between *IGF2BP2* and *IGFBP3* SNPs and clinical‐demographic features of PCOS patients and healthy subjects.

SNP	Group	Genotype	WC (cm)	FBS (mg/dL)	Prolactin (μg/L)	TG (mg/dL)	TC (mg/dL)	HDL‐c (mg/dL)	LDL‐c (mg/dL)
rs1470579	PCOS	AA+AC	100.70 ± 12.88	96.48 ± 14.51	28.37 ± 11.48	132.07 ± 46.81	199.11 ± 15.98	40.40 ± 6.64	110.54 ± 16.75
		CC	107.68 ± 12.54	100.21 ± 23.17	46.32 ± 16.50	177.79 ± 59.76	206.32 ± 17.02	48.43 ± 15.86	109.50 ± 13.76
		*p* Value	0.945	0.504	**<0.001**	0.065	0.585	**0.018**	0.436
	Control	AA+AC	95.02 ± 10.28	93.73 ± 7.27	15.52 ± 4.42	123.92 ± 15.52	190.82 ± 14.86	38.78 ± 3.19	105.47 ± 7.40
		CC	90.07 ± 13.59	98.80 ± 5.92	18.71 ± 5.10	124.53 ± 11.97	179.87 ± 12.82	33.73 ± 2.49	103.53 ± 6.10
		*p* Value	0.530	**0.037**	0.880	0.816	**0.007**	0.724	0.588
rs11705701	PCOS	GG + GA	101.98 ± 13.46	96.72 ± 13.97	30.96 ± 13.47	139.77 ± 36.21	200.80 ± 16.50	40.69 ± 6.72	105.51 ± 12.06
		AA	103.46 ± 7.35	92.77 ± 7.07	28.16 ± 13.79	151.00 ± 10.43	192.77 ± 12.87	39.37 ± 7.51	114.85 ± 5.68
		*p* Value	0.537	0.513	0.441	**0.016**	0.530	0.454	**<0.001**
	Control	GG + GA	93.81 ± 10.28	94.65 ± 6.82	15.73 ± 4.55	124.28 ± 13.78	189.41 ± 14.97	38.17 ± 3.12	105.86 ± 6.99
		AA	99.10 ± 12.26	91.90 ± 9.53	16.34 ± 4.87	122.15 ± 22.11	190.70 ± 15.60	38.80 ± 5.24	102.05 ± 8.48
		*p* Value	0.630	0.095	0.905	0.334	0.876	0.608	0.228
rs2854744	PCOS	GG + GT	102.63 ± 12.79	96.66 ± 14.20	30.68 ± 13.50	140.54 ± 35.92	200.17 ± 16.69	40.67 ± 6.82	106.64 ± 12.15
		TT	101.10 ± 12.96	100.76 ± 26.71	38.41 ± 17.90	156.19 ± 34.11	202.19 ± 14.85	49.52 ± 17.70	104.71 ± 10.08
		*p* Value	0.849	0.126	0.086	0.573	0.948	0.066	0.618
	Control	GG + GT	94.60 ± 10.11	94.08 ± 7.18	15.76 ± 4.70	124.86 ± 13.59	189.28 ± 14.98	38.17 ± 3.33	106.56 ± 5.14
		TT	93.36 ± 16.46	96.82 ± 7.92	15.49 ± 3.06	115.45 ± 27.69	194.64 ± 15.26	39.27 ± 4.73	104.45 ± 6.39
		*p* Value	0.811	0.585	0.246	0.290	0.708	0.392	0.919

*Note:* Bonferroni correction was applied, and *p* < 0.05 is considered statistically significant (bold *p*‐values).

Abbreviations: FBS, fasting blood sugar; HDL‐c, high‐density lipoprotein‐c; LDL‐c, low‐density lipoprotein‐c; PCOS, polycystic ovarian syndrome; SNP, single nucleotide polymorphism; TC, total cholesterol; TG, triglyceride; WC, waist circumference.

### 3.5 In Silico Findings

**FIGURE 1:**
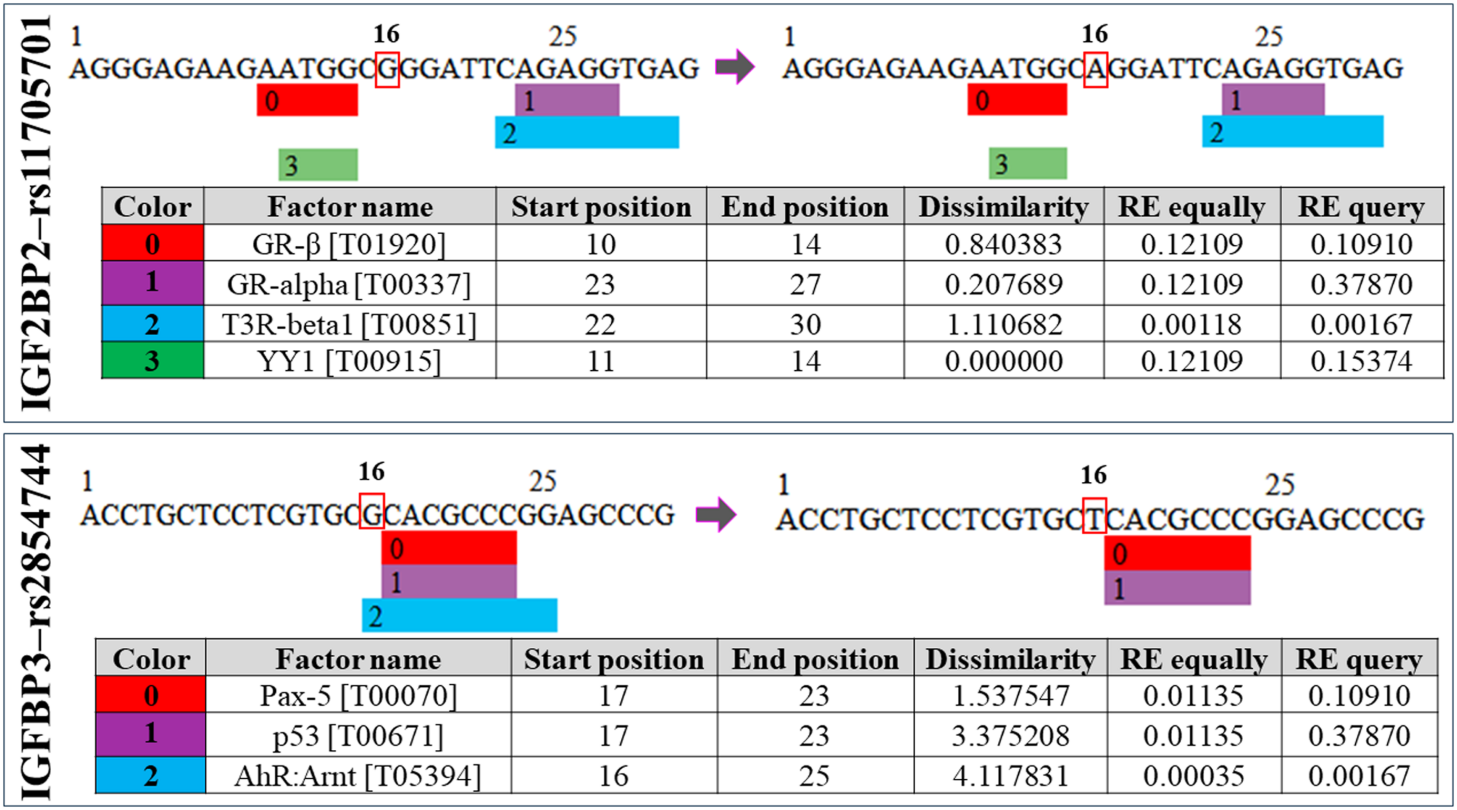
Prediction of the putative transcription factor binding sites in the promoter region of the *IGF2BP2* gene with respect to *rs11705701* and *rs2854744* variations. The red square indicates the SNP position. The random expectation (RE) gives the number of expected occurrences of the match in a random sequence of the same length as the query sequence based on the dissimilarity index. Data are achievable through http://factor.genexplain.com/cgi‐bin/transfac_factor/search.cgi *by* inserting the transcription factor ID. AhR:Arnt, aryl hydrocarbon receptor:Aryl hydrocarbon receptor nuclear translocator; GR‐alpha, glucocorticoid receptor alpha; GR‐beta, glucocorticoid receptor beta; *IGF2BP2*, insulin‐like growth factor 2 mRNA‐binding protein 2; p53, tumor protein 53; Pax‐5, paired box protein 5; rs, reference SNP; SNP, single nucleotide polymorphism; T3R‐beta1, thyroid hormone receptor beta‐1; TFBS, transcription factor binding site; YY1, Yin and Yang 1.

As shown in Figure 1, the analysis of changes in the effect of transcription factors under the influence of rs11705701 and rs2854744 variants in the promoter region of *IGF2BP2* and *IGFBP3* genes showed that the presence of the A allele in the rs11705701 caused no significant changes in binding to the promoter of *IGF2BP2* gene as predicted. Interestingly, in the case of rs2854744, the presence of the G allele created a binding site for a transcription factor (AhR:Arnt [T05394]).

## 4 Discussion

Our study identified a positive association between *IGF2BP2* rs1470579A/C and *IGFBP3* rs2854744G/T and PCOS risk but no association with rs11705701G/A. In addition, statistical analysis showed that the AC/GG/GT, AA/GA/GT, AC/GA/GG, and AC/GA/GT genotype combinations were associated with decreased risk of PCOS. Similarly, the A_rs1470579_A_rs11705701_ haplotype was correlated to the diminished risk of PCOS in our population.

PCOS syndrome patients are more likely to suffer from insulin cell dysfunction and dyslipidemia, contributing to hypertension. PCOS patients often have metabolic and endocrine disorders as well [43]. Tu et al. [44] found that obese, hypertensive, and dyslipidemic patients might also have hyperandrogenism. As discussed below, recent publications have focused mainly on the role of *IGF2BP2* and *IGFBP3* variants in diabetes and cancer risk.

The exact cause of PCOS is unknown, as it manifests as a combination of metabolic, endocrine, environmental, and genetic problems. According to a recent GWAS research, PCOS has been associated with 18 genetic variants on a genome‐wide basis [45, 46, 47]. PCOS‐related regions are mostly connected to metabolic disorders, inflammation, the regulation of insulin signaling, and cancer. As well as offering an early diagnosis of PCOS, susceptibility genes might help prevent obesity, cardiovascular disease, and long‐term T2DM [48]. PCOS females often suffer from insulin resistance [49]. There is a reduction in the amount of insulin that is absorbed by tissues as a result of abnormal insulin receptor function or an excessive level of insulin‐binding antibodies in the blood [50]. PCOS symptoms of insulin resistance and metabolic syndrome are commonly exacerbated by obesity and overweight [51]. Overexpression of some genes related to insulin signaling, including IGF2BP2, has been reported in pancreatic cancer [18]. The rs2854744 G/T (GRCh15.p7, MAF = 0.46) are SNP already linked to the cancer in different populations [52, 53].

As well as sharing traits with other diseases and symptoms, PCOS shares many characteristics with metabolic diseases, inflammation, and insulin signaling disorders. A gene susceptibility test for PCOS may reduce the risk of obesity, cardiovascular disease, and T2DM in the long run [48]. SNPs in genes related to insulin signaling are potential candidates to explain PCOS's clinical manifestations since insulin resistance is a common finding in the disorder [14]. Even though many PCOS women are more likely to develop insulin resistance, poor glucose tolerance, and pancreatic dysfunction, which increases their risk of developing T2DM later in life, there may be fundamental differences in the mechanism of insulin resistance in T2DM and PCOS. Glucose tolerance is reduced, and insulin resistance in diabetic patients causes hyperglycemia. Due to compensatory hyperinsulinemia, steroid hormone metabolism changes, resulting in increased androgen ovarian production, androgen production causing PCOS symptoms [54]. There is a possibility that insulin resistance results from defects in other intracellular insulin receptor signaling, as demonstrated by the observation that female fibroblasts with PCOS show lower tyrosine kinase activity and higher serine kinase activity, both of which are involved in metabolic and mitogenic pathways [55]. In ovaries, insulin binds to IGF receptors due to compensatory hyperinsulinemia or elevated insulin levels. Unlike other tissues, the ovary also uses the inositol–glycan system to signal insulin action rather than receptor tyrosine phosphorylation. However, more research is needed to clarify how insulin binds to the IGF receptor in the ovary [56].

Progesterone aromatization in the ovary can be boosted by IGF‐II, which in turn supports corpus luteum growth and secretion of progesterone and androgen [57]. An in‐depth study showed that PCOS patients' granulosa cells and follicular fluid contain higher concentrations of IGF‐II than those of non‐PCOS patients, as well as GCs [58]. PCOS patients exhibit elevated levels of IGF‐II expression, according to this study. In addition, *IGF‐II* plays a crucial role in the development of PCOS. In PCOS patients, IGF‐II levels were found to be unusually high, which may lead to placental ischemia, hypoxia, and dysplasia, which can result in poor pregnancy outcomes. According to the findings of this study, IGF‐II expression is a separate risk factor that affects pregnancy success in women with PCOS [59]. Interestingly, women with PCOS have shown abnormal *IGFBP2* mRNA levels in their cumulus cells [60]. It has been reported that IGF2BP2 is involved in insulin resistance, tumorigenesis, and lipid metabolism; thus, it can be a possible gene contributing to T2DM, as previously shown by Sargazi et al. [61].

In contrast to our results, in a study conducted by Reddy et al. [17] on 245 cases and 209 controls of Indian ancestry, a negative association between PCOS and genotypes of rs1470579 (OR = 0.71, 95% CI [0.50–1.00], *p* = 0.048). We found that the CC genotype of rs1470579 was positively associated with PCOS risk. This is possibly due to the smaller sample size in our study or the difference in genetic backgrounds among the participants. Moreover, recent studies indicated that the *IGF2BP2* rs1470579 variant might play essential roles in diverse multifactorial diseases, including human metabolic disorders and cancers [15]. Several reports recommended that *IGF2BP2* rs1470579 A>C was related to the risk of T2DM [15]. The SNP has been reported as a risk factor for T2DM in a southeast Iranian population by Sargazi et al. [61] and in a Chinese population by Huang et al. [62], as well as in similar studies [15, 61]. Interestingly, Choi et al. [63] found no significant association between rs1470579 and PCOS. As for the Cikman study, it found no association between LDL levels and the *IGF2BP2* gene, although people carrying the risk allele of the gene had higher LDL levels than those with the AA genotype [64]. According to a different study, female non‐small‐cell lung cancer (NSCLC) patients with the *IGF2BP2* rs1470579 polymorphism showed a statistically significant difference in genotype distribution compared with controls. Control groups had a higher *IGF2BP2* rs1470579 CC genotype prevalence than NSCLC cases [65]. As opposed to that, the *IGF2BP2* rs11705701 SNP was not associated with PCOS in our study, and following the present findings, some studies did not find such an association [15, 61]. Chistiakov et al. [37] found that the rs11705701 A allele was associated with an increased risk of T2DM. The *IGF2BP2* mRNA levels in adipose tissue were higher in nonobese subjects carrying the AA genotype than in other genotypes of *IGF2BP2*. Another study found a strong correlation between *IGF2BP2* rs11705701 and prediabetes in the Chinese population [35]. The Mexican‐American population was at an increased risk of T2DM because of rs11705701, according to a study by Li et al. [36]. Based on the information provided by the variations cataloged in dbSNP (http://ncbi.nlm.nih.gov/SNP/), the rs11705701 allele's frequency differs between populations with MAF = 0.21 in the Chinese individuals, 0.33 in Mexican Americans and 0.38 in Russians. Inconsistency among results may be attributed to differences in MAFs among ethnic groups.

A protein known as IGFBP3 binds to p53 and IGF hormone to induce programmed cell death and regulate IGF hormone activity [66]. *IGFBP‐3* most likely contributes to glucose homeostasis [67]. The retinoid X receptor (RXR)‐transcription factor, which is crucial for maintaining glucose homeostasis, colocalizes with IGFBP3 [68]. Therefore, IGFBP‐3 may affect glucose homeostasis. An essential binding partner of RXR‐ is the peroxisome proliferator‐activated receptor (PPAR), which controls transcription of several glucose and lipid metabolism enzymes. The PPAR receptor is also involved in insulin resistance in women with PCOS [69]. To this date, no experimental evidence has supported a relationship between PCOS and IGFBP‐3 in women with PCOS. In addition, cancer risk increases with a decrease in IGFBP3 and an increase in insulin‐like growth hormone levels [70, 71]. In addition, other factors such as diet, high weight, and physical activity decrease the expression of *IGFBP3* and may have pathological relevance [72, 73].

The rs2854744 is located in the promoter region of the *IGFBP3* gene. Our study showed that homozygous TT genotype might enhance the risk of PCOS. This is consistent with the result of a previous study reporting that the rs2854744 TT genotype was strongly associated with an elevated risk of breast cancer [74]. The AA genotype was associated with a higher *IGFBP3* expression level when adenine was replaced with cytosine in rs2854744. Alternatively, substituting alanine for glycine decreased the binding affinity of IFGs to *IGFBP3* [75].

According to a previous study, PCOS has been associated with low IGFBP‐1 levels and high IGF‐1 levels in the peripheral blood and decidua of women with the condition [76]. Thus, we hypothesized that insulin might also affect other members of the IGFBP family, such as IGF2BP2 and IGF2BP3, thereby influencing IGF levels. In PCOS patients, *IGF2BP2* overexpression is associated with excessive proliferation of the GCs because it preferentially binds to mRNAs with AU‐rich elements (AREs) [13]. This is important because the induction of programmed cell death, called apoptosis, is responsible for the growth of GCs, therefore contributing to the etiology of PCOS [77]. Despite previous results not directly linking IGF2BP2 and IGFBP3 to PCOS etiology, they suggest that these proteins may regulate IGF2 levels and proliferation of cells. The mechanisms involved, however, require further study.

Known as bHLH‐PAS (basic Helix–Loop–Helix‐Period/ARNT/Single‐minded), aryl hydrocarbon receptors (AHR) and aryl hydrocarbon receptor nuclear translocator (ARNT) are transcription factors belonging to the aryl hydrocarbon family [78]. Adaptive and maladaptive responses can be elicited by AHRs [79]. A growing body of research indicates that the AHR is critical in multiple diseases, including PCOS [80]. Several immune cells express the AHR, including T helper 17 (Th17) cells and regulatory T (Treg) cells [81]. Activation of AHR can augment the production of IL‐22 [82], an inflammatory cytokine, causing cutaneous inflammation, PCOS [83], or Crohn's disease [84]. AHR but not ARNT were found to be more present in the endometrium and myometrium of postmenopausal women on continuous hormone replacement therapy [85]. There was a greater expression of AHR mRNA and a lower expression of ARNT mRNA in endometriotic ovarian cysts compared with healthy ovarian tissues. In certain pathological conditions, such as endometriosis, uterine leiomyomas, and presumably PCOS, mRNA expression of transcription factors AHR and ARNT is altered at select target sites. This suggests that these factors may play a role in the pathogenesis of these diseases [85, 86]. In our study, the frequency of the T allele in the group with PCOS was higher and significant (*p* = 0.031). *IGF2BP2* and *IGFBP3* promoter region variants rs11705701 and rs2854744 affect transcription factor binding capability. Based on this analysis, the AhR:Arnt transcription factor loses its ability to bind to the promoter region of the *IGFBP3* gene when the G to T exchange occurs in terms of the rs2854744 variant. Apparently, the presence of this variant might play a crucial role in the development of this syndrome by preventing this transcription factor from binding.

Our study highlighted the role of *IGF2BP2* and *IGFBP3* variations in the course of PCOS. Yet, we are unsure of the functional effects of these changes on the mRNA levels of these genes in PCOS patients. We believe this is the first study to describe the involvement of *IGF2BP2* and *IGFBP3* gene variants in PCOS pathogenesis. Our study was not without limitations; however, we sampled a relatively small number of subjects, and a larger sample size would have increased the statistical power and generalizability of the findings. A second limitation of this study is that the mRNA levels and activities of *IGF2BP2* and *IGFBP3* were not detected. Although random genotyping of the samples showed 100% accuracy, the genotyping results could be confirmed via sequencing. Moreover, we did not perform a gene–environment interaction analysis to determine the possible interactions between individual SNPs and the environment. The Insulin resistance (HOMA‐IR) index and insulin levels in blood samples of cases and controls were not measured, which is another limitation. Besides genetic biomarkers, lifestyle, geography, and race may all contribute to the current study's findings. The connection between these three SNPs and PCOS can be further explored using a larger cohort of patients from different populations.

## 5 Conclusion

In women with PCOS, *IGF2BP2* rs1470579 and *IGFBP3* rs2854744 were associated with an increased risk of developing the condition. The insulin signaling pathway plays essential roles in insulin signaling and glucose metabolism, contributing to PCOS's pathogenesis. Further research on different races is necessary to determine whether these variants play a role in developing and managing PCOS, a complex disorder influenced by multiple factors.

## Author Contributions

Conceptualization: S.S. Writing—original draft preparation: F.G.K., S.S., M.K. Data analysis: M.M. Writing—review and editing: F.G.K., S.S., A.K. Clinical assessments: M.G. Supervision: S.S., F.M. All authors have read and agreed to the published version of the manuscript.

## Ethics Statement

All procedures used in studies involving human subjects complied with the institutional and/or national research committee's ethical requirements, as well as the 1964 Helsinki Statement and its subsequent revisions or comparable ethical standards. The ethics committee of Zahedan University of Medical Sciences approved the study procedure (Ethical code: IR.ZAUMS.REC.1401.288), and the webpage of the ethical approval is available at https://ethics.research.ac.ir/ProposalCertificateEn.php?id=293127&Print=true&NoPrintHeader=true&NoPrintFooter=true&NoPrintPageBorder=true&LetterPrint=true.

## Consent

Participate: Written consent was obtained from all participants.

## Conflicts of Interest

The authors declare no conflicts of interest.

## Supporting information


Appendix S1.

## Data Availability

The data presented in this manuscript will be available by the corresponding author upon reasonable request.
