# Supplementary material for: IGF2BP2 and IGFBP3 Genotypes, Haplotypes, and Genetic Models Studies in Polycystic Ovary Syndrome
Source: J Clin Lab Anal. 2024 Mar 11;38(5):e25021. doi: 10.1002/jcla.25021 (PMC10959184; doi:10.1002/jcla.25021)
Supplement: Supplementary file 1 — Appendix S1. [file JCLA-38-e25021-s001.docx]

**Supplementary Table.1** The sequence of primers used for genotyping *IGF2BP3* polymorphism and *IGF2BP2* polymorphisms.

| **SNP** | **Primers** | **Sequence (5′ to 3′)** | **Annealing Temperature** |
| --- | --- | --- | --- |
| rs11705701 | FO | GGGAAACGAACGCCGAGGGG | 65℃ |
|  | RO | CCTACATTCGTGACTTCTCCAC |  |
|  | Fi (G-allele) | GCCTCCTCACCTCTGAATGCC |  |
|  | Fi (A-allele) | GCCTCCTCACCTCTGAATGCT |  |
| rs1470579 | F | CCTAATTTGATTTTGAGTTTCC | 56 ℃ |
|  | R | CAGGGGTAGATGATGTAAGTGG |  |
| rs2854744 | F | CGGGTCGGTGCAGGCAC | 60℃ |
|  | R | GCTGTATGCCAGTTTCCCCG |  |

FO: Forward outer; RO: Reverse outer; FI: Forward inner; F: Forward; R: Reverse.

**Supplementary Table.2** PCR conditions for genotyping SNPs via ARMS-PCR and PCR-RFLP techniques.

| **Polymorphism** | **Cycle** | **Temperature (ºC)** | **Time (min/sec)** | **Number of cycles** |
| --- | --- | --- | --- | --- |
| rs11705701 | Initial Denaturation  Denaturation Annealing  Extension  Final extension | 95  94  65  72  72 | 5 min  30 sec  30 sec  30 sec  5 min | 35 |
| rs1470579 | Initial Denaturation  Denaturation Annealing  Extension  Final extension | 95  94  56  72  72 | 5 min  30 sec  30 sec  30 sec  5 min | 28 |
| rs2854744 | Initial Denaturation  Denaturation Annealing  Extension | 95  94  60  72 | 5 min  30 sec  15 sec  15 sec | 31 |

**Supplementary Table 3** Allelic and genotypic distribution of *IGF2BP2* and *IGFBP3* polymorphisms.

| **SNP** | **PCOS, n(%)** | **Control, n(%)** | **Genetic model** | **OR (95%CI)^*^** | ***p-value^*^*** |
| --- | --- | --- | --- | --- | --- |
| rs1470579 |  | | | | |
| AA | 59 (39.3) | 81 (55.1) |  | 1 [ reference]  **-** | |
| AC | 63 (42.0) | 51 (34.7) | Codominant 1 | 1.63 (0.95-2.82) | 0.077 |
| CC | 28 (18.7) | 15 (10.2) | Codominant 2 | 3.57 (1.63-7.84) | **0.002** |
|  |  |  | Dominant | 2.08 (1.28-3.38) | **0.003** |
|  |  |  | Recessive | 1.79 (0.88-3.65) | 0.108 |
|  |  |  | Over dominant | 1.31 (0.80-2.13) | 0.282 |
| A | 181 (60.4) | 213 (72.4) | Allelic | 1 [ reference] | |
| C | 119 (39.6) | 81 (27.6) | Allelic | 1.97 (1.37-2.84) | **<0.001** |
| rs11705701 |  | | | | |
| GG | 83 (57.2) | 68 (45.9) |  | 1 [ reference] | |
| GA | 49 (33.8) | 59 (39.9) | Codominant 1 | 0.56 (0.25-1.29) | 0.175 |
| AA | 13 (9.0) | 21 (14.2) | Codominant 2 | 0.52 (0.24-1.14) | 0.103 |
|  |  |  | Dominant | 0.66 (0.40-1.08) | 0.100 |
|  |  |  | Recessive | 0.65 (0.29-1.47) | 0.306 |
|  |  |  | Over dominant | 0.75 (0.45-1.26) | 0.277 |
| G | 215 (74.1) | 195 (65.9) | Allelic | 1 [ reference] | |
| A | 75 (25.9) | 101 (34.1) | Allelic | 0.70 (0.48-1.03) | 0.071 |
| rs2854744 |  | | | | |
| GG | 54 | 70 |  | 1 [ reference] | |
| GT | 74 | 69 | Codominant 1 | 1.39 (0.84-2.29) | 0.201 |
| TT | 21 | 11 | Codominant 2 | 2.54 (1.09-5.87) | **0.030** |
|  |  |  | Dominant | 1.55 (0.96-2.52) | 0.073 |
|  |  |  | Recessive | 2.14 (0.96-4.75) | 0.062 |
|  |  |  | Over dominant | 1.16 (0.72-1.86) | 0.550 |
| G | 182 (61.1) | 209 (69.6) | Allelic | 1 [ reference] | |
| T | 116 (38.9) | 91 (30.4) | Allelic | 1.46 (1.03-2.05) | **0.031** |

*PCOS: Polycystic ovarian syndrome; SNP, Single-nucleotide polymorphism; CI, confidence interval; OR, odds ratio. *p-value and OR (95%CI) were adjusted for body mass index (BMI) and age. Codominant 1 and Codominant 2 indicate the heterozygous and homozygous codominant models, respectively. Bonferroni correction was applied. p<0.05 is considered statistically significant.*


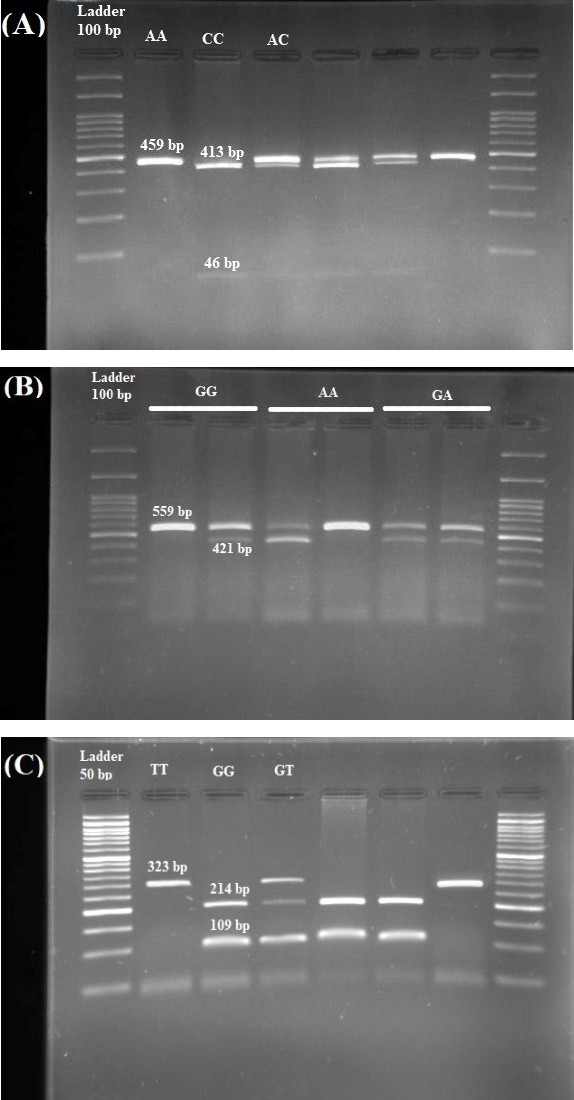


**Supplementary Fig.1** Gel electrophoretic patterns for the PCR products of genotyping *IGF2BP2* rs1470579 (A), *IGF2BP2* 11705701 (B), and *IGFBP3* rs2854744 (C) polymorphisms.


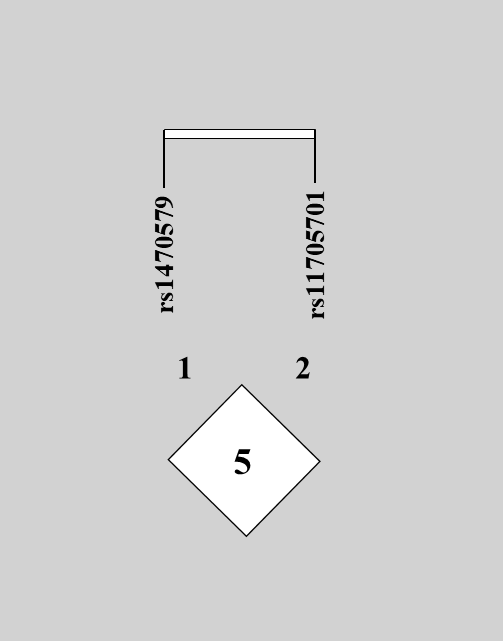


**Supplementary Fig.2** Pairwise LD analysis of *IGF2BP2* rs1470579 and rs11705701 polymorphisms**.**
